# Granulomatous Reactivation during the Course of a Leprosy Infection: Reaction or Relapse

**DOI:** 10.1371/journal.pntd.0000921

**Published:** 2010-12-21

**Authors:** Maria Angela Bianconcini Trindade, Gil Benard, Somei Ura, Cássio Cesar Ghidella, João Carlos Regazzi Avelleira, Francisco Reis Vianna, Alfredo Bolchat Marques, Ben Naafs, Raul Negrão Fleury

**Affiliations:** 1 Health Institute, São Paulo State Health Department, São Paulo, São Paulo, Brazil; 2 LIM 56 and 53, Division of Clinical Dermatology, Clinics Hospital, University of São Paulo, São Paulo, São Paulo, Brazil; 3 Medical Mycology Laboratory, Tropical Medicine Institute, University of São Paulo, São Paulo, São Paulo, Brazil; 4 Lauro de Souza Lima Institute, State Health Department, Bauru, São Paulo, Brazil; 5 Rondonópolis Health Service, State Health Department of Mato Grosso, Rondonópolis, Mato Grosso, Brazil; 6 Curupaiti Health Service, State Health Department of Rio de Janeiro, Curupaiti, Rio de Janeiro, Brazil; 7 Leiden University Medical Centre, Leiden, The Netherlands; Kwame Nkrumah University of Science and Technology (KNUST) School of Medical Sciences, Ghana

## Abstract

**Background:**

Leprosy is a chronic granulomatous infectious disease and is still endemic in many parts of the world. It causes disabilities which are the consequence of nerve damage. This damage is in most cases the result of immunological reactions.

**Objectives:**

To investigate the differences between a type 1 leprosy (reversal) reaction and relapse on using histopathology.

**Methods:**

The histopathological changes in 167 biopsies from 66 leprosy patients were studied. The patients were selected when their sequential biopsies demonstrated either different patterns or maintained the same pattern of granulomatous reaction over more than two years during or after the treatment of leprosy.

**Results:**

In 57 of the patients studied, a reactivation was seen which coincided with a decrease in the bacteriological index (BI), suggesting that this reactivation (reversal reaction or type 1 leprosy reaction) coincides with an effective capacity for bacteriological clearance. In nine patients, an increase of the bacteriologic index (IB) or persistence of solid bacilli occurred during the reactivation, indicating proliferative activity, suggestive of a relapse. The histopathological aspects of the granulomas were similar in both groups.

**Conclusion:**

Bacterioscopy provided the only means to differentiate a reversal reaction from a relapse in patients with granulomatous reactivation. The type 1 leprosy reaction may be considered as a part effective immune reconstitution (reversal, upgrading reaction) or as a mere hypersensitivity reaction (downgrading reaction) in a relapse.

## Author Summary

Leprosy is a serious infectious disease whose treatment still poses some challenges. Patients are usually treated with a combination of antimicrobial drugs called multidrug therapy. Although this treatment is effective against *Mycobacterium leprae*, the bacillus that causes leprosy, patients may develop severe inflammatory reactions during treatment. These reactions may be either attributed to an improvement in the immunological reactivity of the patient along with the treatment, or to relapse of the disease due to the proliferation of remaining bacilli. In certain patients these two conditions may be difficult to differentiate. The present study addresses the histopathology picture of and the *M. leprae* bacilli in sequential biopsies taken from lesions of patients who presented such reactions aiming to improve the differentiation of the two conditions. This is important because these reactions are one of the major causes of the disabilities of the patients with leprosy, and should be treated early and appropriately. Our results show that the histopathology picture alone is not sufficient, and that bacilli's counting is necessary.

## Introduction

Leprosy is a chronic infectious disease caused by *Mycobacterium leprae* and is still endemic in many parts of the world. Circa 250 000 new cases were reported in 2009 [1]. It affects nerves and skin, may cause deformities and may evolve with acute exacerbations. The disease is the result of a granulomatous reaction to bacilli living inside phagocytes; the host therefore depends on cell mediated immunity for bacterial elimination. This response varies in different hosts and therefore gives rise to a clinical spectrum. Two clinical, histopathological and bacteriological stable poles are defined within the spectrum. The pole with high cell mediated immune reactivity to the bacillus is the tuberculoid (TT) pole while the opposite pole with a predominant humoral immune response is the lepromatous (LL) pole. Between these poles, intermediate forms are found; these are immunologically unstable and are called borderline (B): borderline-tuberculoid (BT), borderline-borderline (BB) and borderline-lepromatous (BL) leprosy [2]. A form that can't yet be classified is the indeterminate form (I) [3].

Borderline patients have partial resistance to the bacillus and during the natural course of the disease some bacilli may multiply and induce morphological changes in the granulomatous response. In these cases the granulomas become less compact due to the presence of oedema, with fewer, more dispersed epithelioid cells and infiltrating histiocytes. Jopling referred to this as a downgrading reaction (BB to BL); it was previously described by de Souza, when no treatment was available [4], [5]. It may also occur in borderline patients with irregular treatment or drug resistance, who then move in the leprosy spectrum to the lepromatous pole. The inhibition of the cellular immune response could be due to cell wall antigens such as phenolic glycolipid and/or lipoarabinomanam released by proliferating bacilli [6], [7]. Patients with this evolution (BL to LL) have been classified by Ridley as having the sub-polar lepromatous leprosy (LLsp) [8]. As *M leprae* has a very low replication rate which usually results in incubation time of at least 3 to 7 years, the disease may develop with discrete symptoms; the diagnosis is delayed until clinical manifestations of the downgrading appear. With specific treatment, the bacilli are destroyed and fragmented. However, clearance of the antigens and resolution of the skin lesions occur at a rate that depends largely on the immune resistance of the patient [4], [8], [9].

In this regard, there are borderline patients who during treatment show a clinical reactivation of old lesions or develop new skin lesions with erythema and oedema, suggestive of an upgrading reaction. These lesions tend to regress after a few weeks or months, sometimes even without treatment. However, in many cases biopsies of these lesions still show fragmented bacilli inside nerves, vessels or erector pill muscles or in vacuoles of activated macrophages, which suggests that a downgrading process took place before the upgrading reaction and shift to the tuberculoid pole [9]. The mechanisms underlying such reactions are not clear; it appears that the presence of dead and fragmented bacilli would lead to an improvement of the cell mediated immune response. Although they have originally been described in patients under treatment, they may also occur before or after the treatment, suggesting that they belong to the normal course of a leprosy infection [4], [8], [9], [10].

For better understanding the histopathology of these reactions, effort was made to classify the acute granulomatous tuberculoid reaction found in lesional biopsies of leprosy patients as either a reversal reaction (type 1 reaction or upgrading) or a relapse (with downgrading). We thus hypothesized that a relapse would be defined when the granulomatous reaction was accompanied by an increase or persistence in the bacilloscopy index (BI). On the other hand, a reversal reaction would be defined when the granulomatous reaction was accompanied either by a fast decrease in the BI. For this purpose we analyzed the histopathological changes in patients who show a granulomatous reactivation during or after treatment. Patients with suspected histopathologically reversal reaction and relapse are included, in order to examine the differences between these two states. Relapse has become rare after the introduction of MDT (multidrug-therapy); therefore this study analyzes patients registered between 1987 and 1994, when MDT in Brazil was restricted to a research institution [11].

## Materials and Methods

In this study the leprosy patients included presented with at least one histopathological examination indicating reactivation, determined either by a tuberculoid granulomatous infiltration with a change of classification or by a granulomatous infiltration that persisted for more than two years either during or after treatment. All biopsies were analyzed at the Lauro of Souza Lima Institute (ILSL), SES-SP, Brazil, between 1987 and 1994. Data concerning clinical history were obtained from patients' records from the different centers: 26 from ILSL (Bauru-SP), 16 from the State Institute for Sanitary Dermatology (Curupaiti, RJ) and 24 from the State Center of Dermatology (Rondonópolis, MT). All three sites were referral centers for leprosy patients and followed the guidelines of the Brazilian Program of Leprosy. A total of 179 histopathological examinations from 66 patients were studied: all patients were more than 18 years old, 39 were male, 27 were female. The biopsies were done at the initiation of the treatment and whenever the clinicians suspected of a reaction, which was clinically defined by worsening of the previous lesions or identifying new lesions. Twelve biopsies were excluded because they showed a non-specific inflammatory reaction or no bacilli. All biopsies were identified with a code and processed and analyzed at one center (ILSL, Bauru) by the same pathologists (RNF and MABT), who were not aware of the clinical data at that moment.

The Committee for Ethical Research of the Escola Paulista de Medicina, Universidade Federal de São Paulo approved this study. Informed consent was not necessary because the study was retrospective and no personal identifiers were used.

The Ridley & Jopling histopathological classification was used [2]. The method used for BI determination was counting the bacilli per field according to the criteria established by Ridley & Hilson (1967) [12], using Fite-Faraco staining. This was done in oil immersion, 600× magnification, by examining 25–100 fields, and using a logarithmic scale to score the numbers of bacilli, ranging from 0 to 6. All slides were also stained with haematoxylin-eosin (HE) for histopathology analysis. Granulomatous reactions demonstrating signs of acute inflammation (congestion, oedema, deposit of fibrin, etc.) were classified as Rc (reactional) [8]. Patients with an initial histopathological classification of LL whose later biopsies demonstrated a borderline histopathological picture, were reclassified as LLsp. Patients who histopathologically moved from a TT pattern to BT pattern were then classified as BT. Since a single biopsy may not be sufficient to classify a patient, the definitive classification took the clinical changes through time into account. The TT pole is stable; it is not expected to show any change in clinical, histopathological, and bacteriological examinations. BT patients showed a higher number of skin and nerve lesions than TT patients and the bacterioscopy was usually positive. The bacterioscopy was graded using Ridley's morphological (MI) and bacteriological (BI) indices [12].

## Results

In table 1 the histological patterns of the 66 patients who showed granulomatous reactivation during or after the treatment of leprosy, are recorded, grouped by the diagnosis made at first biopsy: (a) 12 patients were classified as Indeterminate (I); in 11 the histopathology changed to TT or BTRc, only one changed to BB; (b) of the 9 patients classified TT and TRc, 5 continued to be TT, 2 TRc became TT and 2 TT became TRc; (c) of the 17 BT and BTRc patients, 15 remained as BT or BTRc and 2 BT moved down to BB; (d) all 10 patients classified as BB and BBRc became BT or BTRc; (e) of the 10 BL and 8 LLsp patients, 7 BL became BT or BTRc, 3BL became BB or BBRc, while 4 LLsp became BB or BBRc and 4 LLsp became BT or BTRc.

**Table 1:** Evolution of histological patterns from 66 leprosy patients presented reactivation during or after treatment.

1^st^ biopsy*	2^nd^ biopsy	3 ^rd^ biopsy	4^th^ biopsy	≠ patients (≠ with relapse**)
**I**	I	TT	-	1
	TT	-	-	5
	TT	TRc	-	2
	BT	BT	-	1
	BT	BT	BT	1 **(1)**
	BTRc	BT	-	1
	BB	-	-	1 **(1)**
Sub total				12
**TT**	TT	-	-	5
	TRc	-	-	1
	TRc	TT	-	1
Sub total				7
**TRc**	TT	-	-	2
Sub total				2
**BT**	BT	-	-	2 **(2)**
	BT	BT	-	3
	BTRc	-	-	3 **(1)**
	BTRc	BT	-	2 **(2)**
	BB	-	-	2 **(1)**
Sub total				12
**BTRc**	BT	-	-	1
	BT	BTRc	-	1
	BT	BTRc	BT	1
	BTRc	BTRc	BTRc	2
Sub total				5
**BB**	BT	-	-	4
	BTRc	-	-	2
	BB	BT	-	2
Sub total				8
**BBRc**	BTRc	BTRc	-	1
	BB	BT	BT	1
Sub total				2
**BL**	BT	-	-	1
	BT	BTRc	-	1
	BTRc	-	-	1
	BTRc	BT	-	1
	BTRc	BTRc	-	2
	BB	-	-	1
	BBRc	BTRc	-	1
	BBRc	BB	BB	1
	BBRc	BBRc	-	1 **(1)**
Sub total				10
**LLsp**	BT	-	-	2
	BB	-	-	3
	BB	BT	-	1
	BBRc	BTRc	-	1
	BBRc	-	-	1
Sub total				8
TOTAL				66

*Patients were grouped according to the pattern of the first biopsy.

**In parentheses are the 9 cases with relapse. (I) Indeterminate, (TT) tuberculoid-torpid, (TRc) tuberculoid-reactional, (BT) borderline-tuberculoid, (BTRc) borderline-tuberculoid-reactional, (BB) borderline-borderline, (BBRc) borderline-borderline-reactional, (BL) borderline-lepromatosus, (LLsp) lepromatous- subpolar, (-) biopsy not done.

In table 2 the BI of the 66 patients are shown according to the results of the first biopsy: (a) of the 9 patients with a negative BI on the first biopsy, 6 showed a positive BI between 1+ and 4+ on the biopsy taken at the time of reactivation; (b) of the 13 patients with a BI of 1+ on the first biopsy, 7 showed a BI between 1+ and 5+ on the reactivation biopsy; (c) of the 18 patients with a BI of 2+ or 3+ on the first biopsy, 7 showed a BI of 1+ or 2+ on reactivation; (d) of the 26 patients with a BI of 4+, 5+ or 6+ on the first biopsy, 25 showed a BI of 1+ to 5+ on reactivation. Of the other 21 patients, 18 moved to bacterioscopy negative and 3 remained bacterioscopy negative.

**Table 2:** Evolution of the bacilloscopy index from 66 leprosy patients presented reactivation during or after treatment.

1^st^ biopsy*	2^nd^ biopsy	3^rd^ biopsy	4^th^ biopsy	≠ patients (≠ with relapse)**
**Negative BI**	**neg**	-	-	2
	**neg**	**neg**	-	1
	1	**neg**	-	2
	2	-	-	1 **(1)**
	2	**neg**	-	1
	2	2	**neg**	1 **(1)**
	4	1	-	1 **(1)**
Subtotal				9
**BI 1+**	**neg**	-	-	6
	1	-	-	2
	1	**neg**	-	1
	4	-	-	1 **(1)**
	4	3	-	1 **(1)**
	5	-	-	2 **(2)**
Subtotal				13
**BI 2+**	**neg**	-	-	4
	**neg**	**neg**	-	1
	**neg**	**neg**	**neg**	1
	1	-	-	2
	2	2	-	1
Subtotal				9
**BI 3+**	**neg**	-	-	4
	**neg**	**neg**	-	1
	**neg**	1	1	1
	1	-	-	1
	1	**neg**	-	1
	2	2	**neg**	1
Subtotal				9
**BI 4+**	**neg**	3	-	1
	1	-	-	1
	1	1	-	3
	1	1	**neg**	1
	2	1	-	1
	3	-	-	2
	3	1	-	1
	4	-	-	1 **(1)**
	4	1	-	1
Subtotal				12
**BI 5+**	**neg**	-	-	1
	2	-	-	1
	2	**neg**	-	2
	2	1	-	1
	3	-	-	1
	4	-	-	2
	4	1	-	1
	4	1	**neg**	1
Subtotal				10
**BI 6+**	4	-	-	2
	4	4		1 **(1)**
	5	-		1
Subtotal				4
Total				66

*Patients were grouped according to the pattern of first biopsy and in parenthesis are the 9 relapse cases.

**In parentheses are the 9 cases with relapse. Positive BI: bacilloscopy index in+(1 to 6); **neg:** biopsy with absence of bacilli; -: biopsy not done.

Of the 31 patients who had a histopathology showing features of an acute inflammation (Rc), 9 were patients without treatment while 22 patients were on or had already finished treatment; in all cases, the patients had histological patterns ranging from TRc to BBRc, predominating those with BTRc (data not shown).

When the patients were analyzed according to the treatment modality, 9 patients who were treated with dapsone or another monotherapy, but none of the 57 patients who received the WHO MDT regimen, showed an increase in or persistence of their bacilli or the bacterioscopy became positive during the reaction (data not shown).

## Discussion

This study analyzes the histopathological changes of a subset of leprosy patients who showed either a different histopathological pattern on subsequent biopsies or maintained the same pattern of granulomatous infiltration for two years or longer, during or after the treatment of leprosy. They were considered to have a granulomatous reactivation, which includes both reversal reactions and relapses.

The patients who were classified as indeterminate (I) on the first biopsy developed granulomatous reactions that were classified as TT or BT. This occurred regardless of the treatment regimen, with the exception of one patient whose initial biopsy showed a positive bacterioscopy with a very mild inflammatory infiltrate, who subsequently developed BB- leprosy. This suggests that in the more resistant individuals treatment does not necessarily modify the natural course of the disease. Alternatively it may be suggested that the bacteriostatic/cidal action of the drugs would lead to bacilli fragmentation and enhanced antigen exposure, which in turn induced a granulomatous reaction. Analysis of the patients that presented with histopathological acute reactional patterns during treatment showed that the granulomatous reactivations occurred earlier and more frequently among those who had received rifampicin. This is probably a consequence of the rapid bactericidal activity of the drug [13]. This granulomatous reactional (Rc) aspect was also found, albeit less frequently, in the initial biopsy of some patients before treatment; in these cases it was considered to be the result of changes in the immune status of the patients, due to as yet unknown host factors [4], [8], [14], [15].

Patients who at the first biopsy were classified as borderline subsequently showed different histopathological patterns. During the reactivation resulting in granulomatous infiltrations, in some patients the patterns moved towards the tuberculoid pole of the leprosy spectrum, thus presenting an upgrading or reversal reaction. This was more evident in patients initially classified as subpolar lepromatous or borderline lepromatous. Patients with these changes may have gone through a downgrading before treatment, as previously suggested [16]. These reactivations do not necessarily represent a return to the initial situation, because only two histopathological patterns of reactivation were observed (BT and BB); there was no granulomatous reactivation with a BL pattern.

At the tuberculoid pole, some patients showed a reversal reaction while maintaining the same histological presentation, especially the patients classified as BTRc. This was previously reported by Souza Lima & Souza Campos, before the advent of sulphone treatment [5]. Following the start of dapsone treatment it was reported by Opromolla, who suggested that it was caused by episodes of bacterial proliferation (presumably of persisting bacteria) in resistant individuals [17].

In the majority of the patients studied, the episodes of granulomatous reactivation coincided with a decrease in the bacteriological index, suggesting that this reactivation occurs parallel to an effective capacity for bacterial clearance. In contrast, in nine patients there was an increase in the BI during the reactivation episodes (≥2+ relative to the previous BI) or appearance or continuous presence of solid bacilli, indicating bacilli replication [18]–[20]. These reactivations are considered to represent a relapse or a downgrading reaction, probably due to bacteriological resistance or inadequate treatment. These patients had all been treated previously with a single drug regimen; in two of these patients, a change to a MDT regimen resulted in effective reduction in BI in the reactivations/reactions biopsy.

In general the reversal reactions showed a more intense histopathological pattern: more granulomatous with a clearer tuberculoid aspect, more epitheloid cells, usually more grouped than before treatment, suggesting that an active immune reconstitution is taking place. This has been demonstrated in studies showing a more tuberculoid pattern with signs of increased immunological activity with a Th1 response in skin and/or nerves in patients with a reversal or type 1 leprosy reaction when those patients were compared with patients clinically not in reaction. These studies showed enhanced in situ staining for TNF and other pro-inflammatory cytokines such as IFN-γ and IL-12 and for the enzyme nitric oxide synthase in reactional lesions [21], [22].

The same pattern of histopathological changes occurs in patients with AIDS coinfected with *M. leprae* when they start on highly active antiretroviral therapy (HAART). This is considered an immune reconstitution inflammatory syndrome (IRIS). This occurs in some coinfected patients, in whom, due to the AIDS-associated cellular immunesuppression, leprosy remains a latent infection and clinically manifests as type 1 reaction after immune restoration is induced with HAART [23]–[29]. The reactions that appear after the start of leprosy treatment, specially MDT, may represent a similar immune restoration phenomenon [13], [14], [30]. In these cases the bacilli are destroyed by the granulomatous reactivation during the reactional episodes (reversal or type 1 reaction). This is illustrated in the present study by the 57 patients who where on MDT (or in a few cases with an alternative treatment containing rifampicine, the only mycobactericidal drug) and evolved with granulomatous reactional episodes and decrease in the BI [30], [31].

In patients on MDT, episodes of bacillary proliferation of persistent bacilli would be rare and possibly controlled by a similar granulomatous reaction. However, occasionally an increased bacillary load or decreased immune resistance may occur and resemble a relapse. This was observed in patients who had received sulphone mono-therapy exclusively over a long period of time. In these patients, persistent bacilli and/or drug-resistance may give rise to a new episode of bacillary proliferation during or after the treatment; subsequently the host fosters a new granulomatous reaction in a relapse. The granulomatous histopathology of relapses studied here were not different from the histopathology of downgrading type I reactions described by Jopling [4].

Our hypothesis is that the granulomatous reactivations, both the up- and downgrading reactions, can be triggered by live bacilli that multiply. However, in upgrading reaction the granulomatous immune response is effective, leading to destruction of the bacilli and reduction of the BI. In the downgrading reaction, the granulomatous reaction is not as efficient in the control of bacilli multiplication and an increase in the BI can be more easily detected. Thus, the histopathological patterns of granulomatous reactivations are similar and differentiation between reversal and relapses type 1 reactions, are only possible when an increase in BI is seen.
